# Infectious Sclerouveitis in an Immunocompetent Patient: A Probable Case of Simultaneous Ocular Tuberculosis and Toxoplasmosis

**DOI:** 10.7759/cureus.31726

**Published:** 2022-11-21

**Authors:** Wen K Chong, Karen Khoo Kah Kuen, Lam Mun-Wei, Abdul-Salim Ismail, Azhany Yaakub

**Affiliations:** 1 Department of Ophthalmology and Visual Sciences, School of Medical Sciences, Universiti Sains Malaysia, Kota Bharu, MYS; 2 Department of Ophthalmology, Hospital Pulau Pinang, Georgetown, MYS

**Keywords:** immunocompetent, ocular toxoplasmosis, ocular tuberculosis, panuveitis, sclerouveitis

## Abstract

Simultaneous infections of tuberculosis and toxoplasmosis are uncommon in immunocompetent patients. We report a case of a 30-year-old male who presented with right eye redness and blurring of vision for one month. The visual acuities were hand movement and Snellen 20/30 in the right and left eyes, respectively. Panuveitis and scleritis were found in the right eye, together with dense vitritis and a large choroidal lesion that extended from the inferonasal to the superotemporal quadrants. B-scan ultrasonography of the right eye showed a choroidal detachment with scleral thickening and subtenon fluid. His IgM anti-Toxoplasma antibody was detected, and his QuantiFERON Gold testing was positive. Magnetic resonance imaging (MRI) of the orbit revealed an enhancing intraocular mass at the lateral and inferior aspects of the right globe. The diagnosis of right eye sclerouveitis with presumed tuberculosis and toxoplasmosis co-infections was made. He was treated with a course of oral azithromycin and anti-tubercular therapy along with systemic prednisolone and a topical steroid. The treatment reduced the inflammation; however, the patient suffered from a sequela of chronic uveitis with prolonged hypotony. Medical treatment alone may be insufficient in treating severe infective sclerouveitis, hence surgical intervention might be warranted to provide favorable clinical outcomes.

## Introduction

Ocular tuberculosis (TB) and toxoplasmosis often present as infectious uveitides with clinical presentations mimicking each other, posing a diagnostic conundrum. Ocular toxoplasmosis typically presents as focal necrotizing retinochoroiditis with neighboring scars and significant vitreous haze, which may be described as ‘headlights in the fog’ [1]. Apart from the typical presentation, ocular toxoplasmosis can have a variety of other clinical presentations, ranging from granulomatous or non-granulomatous anterior uveitis to posterior segment involvement such as choroidal granulomas, papillitis, or vasculitis [2]. Choroidal involvement, such as posterior uveitis, is the most common ocular presentation of tuberculosis. However, the ocular adnexa, anterior, and posterior segments of the eye can all be affected by diverse tuberculous presentations, which can potentially mimic an ocular tumor [3-5]. The overlapping clinical presentations of different types of infectious uveitides make definite diagnosis challenging. Moreover, the co-infection of multiple causative pathogens further complicates the management of infectious uveitis.

Systemic co-infection of tuberculosis and toxoplasmosis is rather uncommon, and there are few cases reported in endemic countries and immunocompetent patients [6,7]. Primary ocular co-infections with *Mycobacterium* *tuberculosis* and *Toxoplasma gondii* are rare in immunocompetent patients. We report a rare case of sclerouveitis from ocular tuberculosis and toxoplasmosis co-infections in a young, immunocompetent man.

## Case presentation

A 30-year-old male with no known medical history, presented with right eye redness, blurred vision, and pain for a one-month duration. Two days prior to the onset of his symptoms, he described having a dust-like foreign substance enter his right eye, which he was able to remove with irrigation of tap water. Further questioning revealed that he had no previous exposure to individuals with tuberculosis, as well as no contact with cats or animals. Otherwise, he had no history of fever, night sweats, weight loss, or prolonged cough.

**Figure 1:**
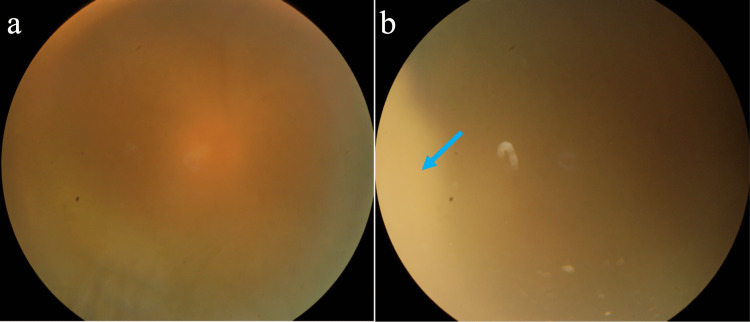
Fundus photo of the right eye. (a) Vitreous haze with a faintly visible optic disc due to dense vitritis. (b) A faint, yellowish-pale, elevated retinochoroidal lesion was seen temporally (cyan arrow).

On examination, he is a medium-built man who stands 170 cm tall and weighs 69 kg. His heart rate was 77 beats per minute, his blood pressure was 128/75 mmHg, and his breathing rate was 15 breaths per minute. Examinations of the neurological, cardiovascular, and pulmonary systems were all normal. There was no lymphadenopathy. His visual acuities were hand movements and Snellen 20/30 in the right and left eyes, respectively. The right eye examination showed severe panuveitis with conjunctival hyperemia, anterior chamber cells 4+, grade 3 vitreous haze, and an intraocular pressure of 6 mmHg. There were no keratic precipitates, hypopyon, iris nodules, or iris atrophy. Fundus examination showed dense vitritis (Figure 1a) and a large, yellowish, elevated, retinochoroidal lesion temporally, extending from 6 to 12 o’clock (Figure 1b). No obvious pigmented chorioretinal scars were seen. The examination of the left eye was otherwise unremarkable. 

**Figure 2:**
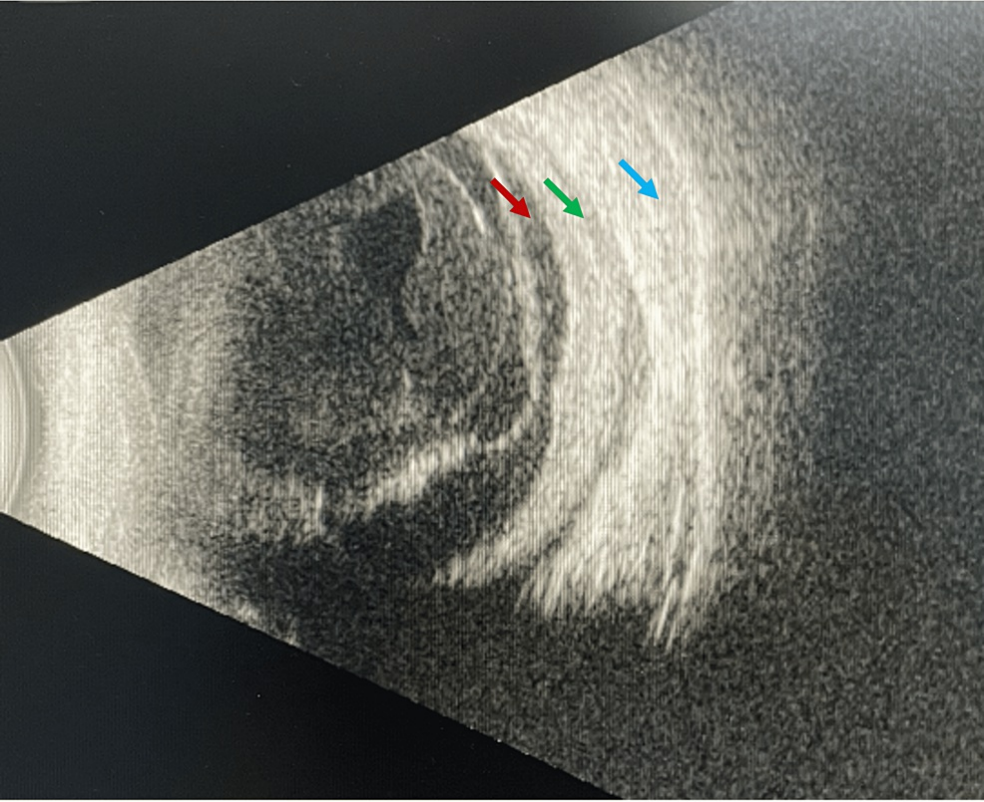
B-scan ultrasonography of the right globe showed retinal detachment (red arrow), thickened and detached choroid (green arrow), and thickened sclera (cyan arrow).

**Figure 3:**
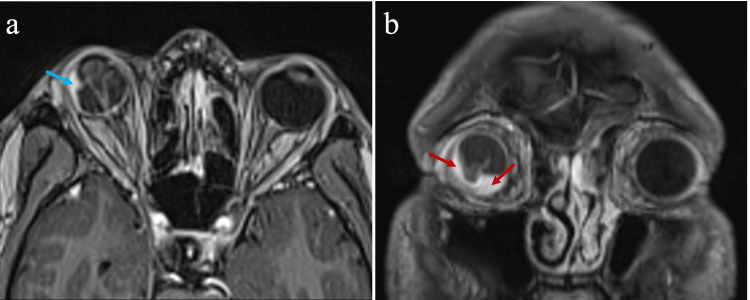
T1 weighted MRI (a) axial section showing enhancing lesion at the temporal region of the right globe (cyan arrow). (b) Coronal section revealed the lesion extending from the temporal to the inferior region of the right globe (red arrow). MRI: magnetic resonance imaging.

The B-scan ultrasonography of the right eye (Figure 2) showed a thickened choroid with choroidal effusion, scleral thickening, and subtenon fluid, corresponding to the lesion seen on fundus examination. An exudative retinal detachment was demonstrated on the B-scan ultrasonography. Contrast-enhanced computed tomography (CT) of the orbit revealed an irregular mass seen in the right lateral aspect of the right globe. There was no intraocular foreign body visualized. Magnetic resonance imaging (MRI) of the orbit (Figure 3) revealed an enhanced intraocular mass at the lateral and inferior aspects of the right globe. 

His whole blood count did not reveal any abnormalities or a notable increase in leucocytes. His routine renal profiles, liver function tests, fasting blood sugar levels, and fasting lipid profiles were all within normal limits. However, his erythrocyte sedimentation rate (ESR) and C-reactive protein (CRP) were elevated at 43 mm/h and 14 mg/L, respectively. The IgM anti-Toxoplasma antibody was detected in his serum. The tuberculin skin test was equivocal with a skin induration of 14 mm in diameter; however, the QuantiFERON Gold testing was positive. There was no lesion seen on the chest x-ray. The findings of other laboratory tests for autoimmune illnesses, the venereal disease research laboratory test (VDRL), and the human immunodeficiency virus (HIV) were all negative. The test for tumor markers was likewise negative. 

Based on the results of the positive anti-Toxoplasma IgM antibody and interferon-gamma release assay laboratory tests, sclerouveitis of the right eye with suspected co-infection of TB and toxoplasmosis was diagnosed. He was co-managed by an infectious disease physician and a pulmonologist. He received a course of oral azithromycin 500 mg once daily for six weeks, an intensive phase of anti-tubercular therapy consisting of daily doses of 300 mg of isoniazid, 600 mg of rifampicin, 1100 mg of ethambutol, and 1600 mg of pyrazinamide for three months, followed by an anti-tubercular maintenance phase consisting of 300 mg of isoniazid and 600 mg of rifampicin for six months. A tapering dose of systemic prednisolone (1 mg/kg) and topical steroids were also prescribed two months after the commencement of anti-tubercular therapy. After two months of therapy, the medicine reduced the right eye's inflammation, although low-grade inflammation continued. The right eye aqueous humor aspirate was then performed and analyzed by polymerase chain reaction (PCR), which turned out to be negative for *M. tuberculosis*. Moreover, the cytology analysis of the aqueous humor was also negative for malignant cells.

Pars plana vitrectomy for diagnostic and therapeutic purposes was offered to the patient; however, it was rejected by the patient. After completion of a six-week course of oral azithromycin and anti-tubercular therapy for nine months in combination with tapering oral prednisolone, ocular inflammation eventually subsided but hypotony persisted. Owing to persistent hypotony, the right eye became phthisis bulbi with the development of white cataracts, band keratopathy, and rubeosis iridis.

## Discussion

Sclerouveitis is commonly seen in systemic vasculitides and inflammatory conditions such as systemic lupus erythematosus (SLE), rheumatoid arthritis, sarcoidosis, Behcet’s disease, and granulomatosis with polyangiitis, but ocular infections such as syphilis, tuberculosis, Lyme’s disease, and toxoplasmosis can also present as sclerouveitis [8,9]. Only one out of 35 individuals with sclerouveitis, as found in our patient, had panuveitis, according to Manandhar et al.'s report of the patterns of uveitis and scleritis [10]. Of all infectious sclerouveitis, tubercular infections were the most common cause, followed by herpes zoster infection and leprosy [10].

Our patient presented with right eye sclerouveitis with panuveitis and was diagnosed with ocular co-infection of tuberculosis and toxoplasmosis with evidence of a positive QuantiFERON-TB Gold test and anti-Toxoplasma IgM antibodies. Both ocular tuberculosis and toxoplasmosis can share a resemblance in terms of clinical manifestations, such as retinochoroiditis and vitritis. To ascertain the underlying etiology of any uveitis case often poses a significant diagnostic as well as therapeutic challenge for clinicians. Diagnosis is primarily made on the basis of clinical features from history and clinical examinations and microbiological and radiological investigations [11]. To diagnose ocular tuberculosis without primary pulmonary disease, evidence of eye involvement demonstrated by granulomatous inflammation and detection of acid-fast bacilli through biopsy is typically required [4]. However, it is often the case that Mycobacteria were not easily found [12]. Since the advent of the PCR test with variable sensitivity and high specificity in identifying the causative organisms, it has become a helpful tool in aiding clinicians to draw a diagnosis. The PCR of our patient’s aqueous humor aspirate was found to be negative for *M.* *tuberculosis,* which could be due to the late timing of sample collection. Aqueous samples were collected three months after the initiation of anti-tubercular therapy, which could have exterminated the mycobacteria. Therefore, if specific investigations can be performed before or shortly after the initiation of treatment, the clinical diagnostic value will be greater. Thence, there is a limitation in this case that these could have been the probable infections as only preliminary tests were positive, and such could have been positive in endemic countries.

Tubercular ocular disease is a great masquerade that gives rise to a wide range of clinical presentations. Cases of ocular tuberculosis masquerading as ocular tumors have been reported [13]. A case of recurrent sclerouveitis due to ocular tuberculosis with a mass within the ocular coat has been reported by Damodaran et al [4]. The diagnosis of ocular tuberculosis was made through the PCR result of the enucleated eye, which showed positive for the *M. tuberculosis* genome of IS6110, despite typical granulomatous inflammation not demonstrated histopathologically [4]. Similarly, an intraocular mass was seen in our patient, as demonstrated by CT and MRI findings. Along with the clinical presentation of sclerouveitis and panuveitis, infective causes were at the top of the list. Primary ocular malignancies or metastases should also be considered and excluded. Kirn et al. reported a case of recalcitrant sclerouveitis secondary to peripheral T-cell lymphoma that responded partially with systemic chemotherapy [14]. Ocular malignancies were considered less likely in our patient due to his young age, unremarkable medical history, systemic examination, and absence of malignant cells in aqueous humor aspirate. A biopsy of the ocular lesion is ultimately needed for a definitive diagnosis, which could be of technical difficulty. The risk of globe perforation is increased when the friable, inflamed ocular tissue is biopsied. 

The use of corticosteroids and immunosuppressive agents in non-infectious uveitis is the cornerstone of treatment to modulate the inflammatory response. However, the role of immunomodulation in the treatment of ocular tuberculosis is still debatable. A recent meta-analysis revealed that favorable outcomes were observed in approximately 85% of patients with tubercular uveitis treated with anti-tubercular therapies alone, with or without systemic steroids or immunomodulators [15]. Thence, no conclusion about which regimen is recommended to control ocular inflammation could be drawn. Meanwhile, for visually threatening ocular toxoplasmosis, the mainstay of treatment includes systemic anti-Toxoplasma agents and oral corticosteroids. There is, however, no consensus regarding which anti-Toxoplasma antibiotics are the best treatment regimen. In a network meta-analysis by Zhang et al., comparable efficacy was found in the resolution of vitreous inflammation of ocular toxoplasmosis when treated with clindamycin, azithromycin, and trimethoprim-sulfamethoxazole compared to that of pyrimethamine-sulfadiazine [16].

Inadequate response to medical treatment was observed in our patient, similar to the case reported by Agarwal et al [11]. Pars plana vitrectomy helps in the clearance of vitreous opacity, which enables detailed fundus examination and the acquisition of a vitreous sample for diagnostic purposes [17,18]. Vitrectomy has also been proven efficacious in reducing antigenic load and inflammatory factors within the vitreous [11,17,18]. There was a significant improvement in visual acuity reported in patients with infectious, non-infectious, and unidentified uveitis groups after vitrectomy [17]. A diagnostic and therapeutic impasse was encountered in our patient due to reluctance for further surgical intervention, which we deemed could have been beneficial.

## Conclusions

It is challenging for the clinician to make a definite diagnosis of co-infection of ocular tuberculosis and toxoplasmosis without evidence of pathogen isolation. The majority of presumed ocular tuberculosis and toxoplasmosis diagnoses are mostly based on clinical symptoms and ancillary investigations globally, especially in resource-limited settings. Albeit timely empirical medical therapies have been administered, an inadequate response with devastating consequences due to a prolonged course of inflammation is possible. The choice of other therapeutic modalities should be considered.
